# A polyepigenetic glucocorticoid exposure score and HPA axis-related DNA methylation are associated with gestational epigenetic aging

**DOI:** 10.1080/15592294.2025.2471129

**Published:** 2025-02-25

**Authors:** Allison A. Appleton

**Affiliations:** Department of Epidemiology and Biostatistics, University at Albany College of Integrated Health Sciences, Rensselaer, NY, USA

**Keywords:** Gestational epigenetic age deviation, polyepigenetic glucocorticoid exposure score, HPA-axis DNA methylation, developmental origins of health and disease

## Abstract

Gestational epigenetic aging (GEA) is a novel approach for characterizing associations between prenatal exposures and postnatal risks. Psychosocial adversity in pregnancy may influence GEA, but the molecular mechanisms are not well understood. DNA methylation to glucocorticoid regulation and hypothalamic-pituitary-adrenal (HPA) axis genes are implicated but have not been fully examined in association with GEA. This study investigated whether a polyepigenetic glucocorticoid exposure score (PGES) and HPA axis gene (*NR3C1, HSD11B2, FKBP5*) methylation were associated with GEA, and whether associations were sex-specific. Participants were from a prospective cohort of racial/ethnic diverse and socially disadvantaged pregnant women and infants (*n* = 200). DNA methylation variables were estimated using umbilical cord blood. PGES was derived with CpGs shown to be sensitive to synthetic dexamethasone exposure. *NR3C1*, *HSD11B2*, and *FKBP5* methylation was summarized via factor analysis. We found that PGES (β = -1.12, SE = 0.47, *p* = 0.02) and several *NR3C1* and *FKBP5* factor scores were associated with decelerated GEA (all *p* < 0.05). A significant sex interaction was observed for *FKBP5* factor score 3 (β = -0.34, SE = 0.15, *p* = 0.02) suggesting decelerated GEA for males but not females. This study showed that glucocorticoid regulation-related DNA methylation was associated with a decelerated aging phenotype at birth that might indicate a neonatal risk.

## Introduction

The gestational environment and developmental changes occurring during pregnancy can provide a basis for lifelong health and functioning for the offspring [1]. The past few decades of Developmental Origins of Health and Disease (DOHaD) research has illustrated the sensitivity of the prenatal milieu, with a range of exposures in pregnancy contributing to offspring health over the life course [1–4]. Due to the epigenome’s malleability during gestation, epigenetic changes are plausible linking mechanisms between prenatal exposures and offspring phenotype, with supporting evidence accumulating from traditional epigenome wide association and candidate gene examinations [3], as well as from newer applications like epigenetic aging [5,6]. Gestational epigenetic aging (GEA) is a novel approach for characterizing impacts of fetal exposures on postnatal risks, which involves estimating the degree to which biologic age (estimated with DNA methylation at subsets of CpGs from birth tissues like placenta and umbilical cord blood) is accelerated (older) or decelerated (younger) than expected given the chronologic age typically measured via ultrasound [7].

Psychosocial stress during pregnancy may contribute to gestational epigenetic alterations to hypothalamus-pituitary-adrenal (HPA) axis genes like *NR3C1, HSD11B2*, and *FKBP5*, which may in turn influence infant health and neurodevelopmental risks for the neonate [8–15]. Emerging work similarly suggests that deviations in GEA may be mechanism linking maternal stress and depression during pregnancy with offspring outcomes. For example, a set of cohort studies have found depression and psychosocial adversities during pregnancy to be associated with gestational epigenetic age deceleration among infants in independent samples [16–19], and one study observed decelerated GEA influenced child behavioral functioning at age 3.7 years [16]. Where accelerated epigenetic aging may signify older biologic age and health risk among adults [6], neonatal decelerated epigenetic aging may indicate a more premature physiology than would be expected given chronological age at delivery [20]. Thus, deviations in GEA may help explain health and developmental risks for infants related to maternal psychosocial stress in pregnancy.

While the population-based evidence is accumulating suggesting a link between maternal depression and stress with GEA, the molecular determinants of GEA are not well understood. It is plausible that glucocorticoid and HPA-axis pathway dysregulation could be involved. Provençal and colleagues [21] derived a polyepigenetic glucocorticoid exposure score (PGES) that quantifies methylation extent among a set of CpGs found to be sensitive to exogenous glucocorticoid exposure and contributed to long-lasting changes in DNA methylation in progenitor cells; the PGES also discriminates risk among pregnant women and children. For example, in a prospective cohort of 810 pregnancies, prenatal depression and anxiety were associated with lower levels of PGES in umbilical cord blood [21]; PGES at birth in turn was associated with increased severity of child mental and behavioral problems at ages 7–10 years [22]. To date, only one study has examined the relationship between PGES and GEA. In a sample of 83 post-partum women, higher levels of umbilical cord derived PGES was associated with decelerated gestational epigenetic aging among infants, with sex-specific patterning in GEA suggesting differential susceptibility to PGES for males and females [23]. While this small study provides initial evidence that PGES can influence GEA, replication work in an independent sample is warranted. Moreover, while the PGES includes molecular marks sensitive to exogenous glucocorticoid exposure, it does not include genomic locations related to the HPA-axis pathway. This is surprising given the large evidence base linking methylation extent to HPA-related genes such as *NR3C1, HSD11B2*, and *FKBP5* to maternal mental health in pregnancy and offspring phenotype [8]. Thus, extension of this initial work into the molecular determinants of GEA that explicitly considers HPA-axis related CpG sites is warranted.

The present study has three aims. First, we endeavored to replicate the association between PGES and GEA [23] in an independent sample. As was observed in this prior work, we hypothesized that higher gestational PGES scores would be associated with decelerated GEA. We tested this hypothesis among a larger and more diverse study population than has previously been considered. Second, we examined whether patterning in gestational methylation extent to a set of HPA axis genes would be associated with deviations in GEA. We considered three genes indicative of HPA axis and glucocorticoid regulation that prior research has found to be related to maternal psychosocial adversity and infant risk: *NR3C1, FKBP5, HSD11B2*. Third, as prior work has found sex-specific associations [11,23], we examined whether associations between PGES and HPA-related DNA methylation with GEA was patterned by infant sex. To our knowledge, this study is the first to consider whether a range of glucocorticoid related DNA methylation markers may influence epigenetic aging signatures at birth.

## Methods

### Study population

The Albany Infant and Mother Study (AIMS) is a prospective cohort study of pregnant people and their infants born at Albany Medical Center (Albany, NY, USA) and has been described in several previous publications [18,24–27]. Briefly, English speaking individuals who were pregnant with one baby and between the ages 18–40 years of age were eligible to participate. Following the informed consent process, the enrollment visit (occurring around 28 weeks gestation) included questionnaire administration and biospecimen collection to collect pre-pregnancy and gestational exposure information. At the birth, umbilical cord blood samples were collected, and infant anthropometry was assessed in the delivery room by trained clinicians using standard protocols and instrumentation. After the birth, study physicians abstracted medical records for information on pregnancy morbidity, delivery factors, and birth outcomes. Three-hundred pregnant people were screened, 272 were eligible and enrolled, and 204 provided umbilical cord blood samples. Three outliers (i.e., those whose DNA methylation predicted gestational age was greater than three standard deviations from the mean of actual gestational age) were removed from the sample. Here, we present a complete case analysis of 200 individuals who had available epigenetic and covariate data. The Albany Infant and Mother Study was approved by Institutional Review Boards at the University at Albany State University of New York and Albany Medical Center.

### Measures

#### DNA methylation

DNA from umbilical cord blood samples was extracted and methylation was assessed via the Illumina EPIC Infinium array (Illumina, San Diego, CA) [28]. As described in our previous AIMS publications [18,27], we undertook the standard statistical processing pipeline for analyzing EPIC array data, and followed established protocols (e.g., extractions from raw files using the *minfi* R package [29], functional normalization, Beta Mixture Quantile Dilation (BMIQ) [30], ComBAT adjustment [31,32]. This process yielded β values (a ratio of methylated/unmethylated probes). We also estimated cell type proportions [33]. We used this processed DNA methylation data to derive the gestational epigenetic age clock metric [7], the glucocorticoid epigenetic score [23], and the gestational HPA-related methylation variables [27], each described in turn below.

##### Gestational epigenetic age deviation

As described previously [18], and in order to facilitate comparisons to other recent research in this area [16–18,23], we estimated gestational epigenetic age deviation with the Knight method [7] which was developed specifically for umbilical cord blood derived DNA. The Knight clock measure is a weighted average of methylation extent at 148 CpGs; we calculated GEA with the 141 probes covered by the EPIC array [18]. Previous research that has shown that DNA methylation age estimates derived with the EPIC array were robust to missing probes compared to other platforms [34]. Sensitivity analyses described previously likewise showed GEA was robust to the removal of the seven probes in the original Knight clock that are not included on the EPIC array [18]. We examined the GEA residual variable, which represents the difference between epigenetic gestational age and clinically estimated gestational age (via ultrasound), calculated as the residual from a regression analysis where the epigenetic age is regressed against the clinical gestational age. Positive and negative deviations to residual scores can indicate acceleration and deceleration respectively.

##### Polyepigenetic Glucocorticoid Exposure Score (PGES)

The PGES is a set of 24 CpGs identified in prior work with fetal hippocampal progenitor cells and peripheral blood where lasting changes in DNA methylation were observed in response to synthetic dexamethasone exposure [21]. This score was originally derived with weights corresponding to the magnitude of methylation change at each component CpG [21]. As the weights are tissue specific [19], unweighted PGES scores have also been derived and been examined across other tissues including buccal cells [19] and umbilical cord blood [23]. Here, using umbilical cord derived DNA, we calculated an unweighted PGES score as a simple arithmetic average of methylation extent across component CpGs [23]. Methylation information was available at 23 of the 24 glucocorticoid sensitive CpGs [19,23] and were included in this unweighted PGES.

##### Hypothalamic pituitary adrenal axis epigenetic scores

We considered DNA methylation for three HPA axis genes: *NR3C1* (85 CpGs), *HSD11B2* (23 CpGs), and *FKBP5* (50 CpGs). All CpGs for each gene that were measured in the EPIC array were included in analysis. There was no overlap in the CpGs for these genes with those included in the PGES. We used factor analysis to summarize DNA methylation separately for *NR3C1*, *HSD11B2*, and *FKBP5*. This approach helps to reduce the dimensionality of large data, helps prevent type I error, and is often applied in gene-expression, and microarray analyses [27,35,36]. As described in our prior work [27], we conducted an exploratory factor analysis to extract factors for each gene, and then conducted a parallel analysis incorporating a Monte Carlo simulation to determine the number of factor to retain [37,38]. The parallel analysis is considered a best-practice for determining the number of factors to retain [38]. This process resulted in six *NR3C1* factors, and seven factors each for *HSD11B2*, and *FKBP5*. The fit statistics indicated good model fit for each gene (RMSEA_*NR3C1*_ = 0.06; RMSEA_*HSD11B2*_ = 0.05; RMSEA_*FKBP5*_ = 0.06). Supplemental file 1 lists descriptive data for the HPA axis epigenetic scores, where loadings largely demarcated factors by genomic location and methylation extent. Proportion of variance explained for each factor was moderate, ranging from 0.36 to 0.07. For each retained factor, DNA methylation was averaged for the all the CpGs that loaded on that factor.

##### Covariates

Demographic, maternal health, and infant factors were controlled for in analysis. Demographics were self-reported maternal age, race/ethnicity (white and not-Hispanic; Black/Hispanic/other) and education attainment (≤high school degree/GED; >high school degree). Maternal health factors were assessed via self-report at the pregnancy enrollment visit and from medical records. Self-reported factors included pre-pregnancy body mass index (kg/m [2], smoked during pregnancy (yes/no), depressive symptoms, and diet. The Edinburgh Postnatal Depression Scale (EPDS; α = 0.87) [39] measured depressive symptoms in pregnancy. Diet was measured with an abbreviated Food Frequency Questionnaire [40,41], and a western dietary pattern score was calculated indicating the frequency of eating western-type foods (i.e., red/processed meats, high-fat dairy, refined/processed grains, soda) [25–27]. Medical record sourced covariates included parity (nulliparous/not nulliparous), pregnancy complications, and infant attributes. Pregnancy complications was a count of the following conditions during the focal pregnancy: gestational diabetes, preeclampsia, eclampsia, placental abnormalities (abruption, previa, accreta, marginal bleed), bacterial infections (Group B Streptococcus, Chorioamnionitis), and PPROM. Infant covariate data was sex (male/female) and gestational age at delivery (weeks; abstracted from medical records; estimated from ultrasound during prenatal care visits).

### Analysis plan

First, we calculated descriptive statistics for study variables for the full sample and according to infant sex. Independent *t* and χ^2^ tests considered sex differences for continuous and dichotomous variables respectively. Then, we examined Pearson’s correlations among all epigenetic variables (GEA, PGES, and the HPA methylation scores), while controlling for cell type proportions. These correlations illustrated the degree to which the epigenetic predictors were independent from one another, as well as highlighted the bivariate associations with GEA. While the GEA was not correlated with cell type proportions, PGES and some HPA methylation scores were correlated with cell type (data not shown); therefore, we controlled for cell type heterogeneity in analysis. Next, we fit a set of multivariable linear regression models testing the main effect associations between PGES and each HPA methylation score with GEA, while controlling for cell type proportions and study covariates. Sex differences were assessed in two ways. The multivariate models were stratified by infant sex to determine if associations for PGES and HPA methylation scores with GEA were different for males and females. For sex-specific associations that were significant in stratified analyses, additional models were built that included a multiplicative interaction term for that epigenetic predictor with infant sex and examined in association with GEA, controlled for study covariates. For the multivariate models, PGES and HPA methylation scores were standardized (mean = 0, standard deviation = 1) for interpretability. Statistical significance was determined by p-values less than 0.05.

## Results

Table 1 lists participant characteristics. Overall, women were on average 29 years old during pregnancy, 41% were from a racial/ethnic minority group, and 34% reported high school or less as the highest level of education attained. Eleven percent of women smoked during pregnancy, 25.5% were nulliparous, the average pre-pregnancy BMI was 29.02 (SD = 8.53), and the depressive symptoms score was 8.70 (SD = 5.33). Women experienced on average less than one pregnancy complication (range 1–4; each condition was < 10% prevalence) and had western diet scores of 39.70 (SD = 14.60). Infants were largely born at term (*M* = 39.04 weeks, SD = 1.65). There was an even split of male and female infants in the sample. There were no significant differences in maternal characteristics according to infant sex (all *p* > 0.05), though smoking during pregnancy was marginally more prevalent among participants with male (15.15%) as compared to female infants (6.93%; *p* = 0.06).

**Table 1.** Albany infant and mother study participant characteristics*.

	Full sample (*n* = 200)	Males (*n* = 99)	Females (*n* = 101)	p^^^
**Maternal characteristics**				
Race, white/not Hispanic, % (n)	59.00 (118)	60.61 (60)	57.43 (58)	0.65
Race, not white and/or Hispanic, % (n)	41.00 (82)	39.39 (39)	42.57 (43)	
Education, high school or less, % (n)	34.50 (69)	37.37 (37)	31.68 (32)	0.40
Education, more than high school, %, (n)	65.50 (131)	62.30 (62)	68.32 (69)	
Smoked during pregnancy, yes, % (n)	11.00 (22)	15.15 (15)	6.93 (7)	0.06
Smoking during pregnancy, no, % (n)	89.00 (178)	84.85 (84)	93.07 (94)	
Nulliparous, yes, % (n)	25.50 (51)	26.26 (26)	24.75 (24)	0.80
Nulliparous, no, % (n)	74.50 (149)	73.37 (73)	75.25 (76)	
Age, years,	28.63 (5.51)	28.62 (5.72)	28.65 (5.33)	0.48
Depressive symptoms, sum score	8.70 (5.33)	9.10 (5.26)	8.30 (5.41)	0.29
Pre-pregnancy BMI	29.02 (8.53)	28.62 (7.86)	29.42 (9.17)	0.51
Pregnancy complications, index	0.72 (0.97)	0.65 (0.96)	0.78 (0.99)	0.33
Western diet, sum score	39.70 (14.60)	40.98 (13.97)	38.47 (15.16)	0.22
Gestational age at delivery, weeks	39.04 (1.65)	39.04 (1.55)	39.04 (1.74)	0.99
**Epigenetic characteristics**^**+**^				
DNAm gestational age, weeks	39.82 (1.66)	39.68 (1.46)	39.95 (1.83)	0.25
GEA, residual	−0.09 (1.03)	−0.18 (1.08)	0.01 (0.98)	0.19
PGES	0.35 (0.05)	0.35 (0.04)	0.35 (0.06)	0.98
*NR3C1* − 1	0.72 (0.02)	0.72 (0.02)	0.72 (0.02)	0.85
*NR3C1* − 2	0.75 (0.01)	0.74 (0.02)	0.75 (0.01)	0.08
*NR3C1* − 3	0.06 (0.003)	0.06 (0.003)	0.06 (0.003)	0.07
*NR3C1* − 4	0.68 (0.02)	0.68 (0.02)	0.68 (0.02)	0.31
*NR3C1* − 5	0.58 (0.02)	0.58 (0.02)	0.57 (0.02)	0.06
*NR3C1* − 6	0.61 (0.02)	0.61 (0.02)	0.61 (0.02)	0.86
*HSD11B2* − 1	0.02 (0.002)	0.02 (0.002)	0.02 (0.002)	0.49
*HSD11B2* − 2	0.63 (0.04)	0.63 (0.04)	0.63 (0.04)	0.63
*HSD11B2* − 3	0.59 (0.02)	0.58 (0.02)	0.60 (0.02)	<0.001
*HSD11B2* − 4	0.39 (0.01)	0.38 (0.01)	0.39 (0.01)	0.06
*HSD11B2* − 5	0.04 (0.01)	0.04 (0.01)	0.04 (0.01)	0.87
*HSD11B2* − 6	0.01 (0.001)	0.01 (0.001)	0.01 (0.002)	0.29
*HSD11B2* − 7	0.90 (0.01)	0.90 (0.01)	0.89 (0.01)	0.06
*FKBP5* − 1	0.84 (0.02)	0.84 (0.02)	0.84 (0.02)	0.97
*FKBP5* − 2	0.39 (0.05)	0.39 (0.04)	0.39 (0.06)	0.51
*FKBP5* − 3	0.52 (0.02)	0.52 (0.02)	0.52 (0.02)	0.23
*FKBP5* − 4	0.02 (0.002)	0.01 (0.002)	0.02 (0.002)	0.10
*FKBP5* − 5	0.39 (0.07)	0.39 (0.08)	0.39 (0.07)	0.98
*FKBP5* − 6	0.82 (0.02)	0.82 (0.01)	0.81 (0.02)	0.14
*FKBP5* − 7	0.48 (0.01)	0.48 (0.01)	0.48 (0.01)	0.37

*Cell entries are means (standard deviations) unless otherwise specified. ^**+**^Epigenetic characteristics GEA, PGES, *NR3C1, HSD11B2*, and *FKBP5* scores represent proportion methylated. ^^^ p-value corresponds to independent *t*- and χ^2^ tests for continuous and categorical variables respectively. Abbreviations: DNAm=DNA methylation; GEA=Gestational epigenetic aging; PGES=Polygenetic glucocorticoid exposure score.

The average DNA methylation predicted gestational age was 39.82 weeks (SD = 1.66), and measured gestational age was 39.04 weeks (SD = 1.65). There was a significant positive correlation between gestational epigenetic age and clinically assessed gestational age (*r* = 0.73, *p* < 0.001). The mean residualized GEA in the analytic sample was −0.09 (SD = 1.03). The CpGs comprising the PGES were on average 35% methylated (range 21–53%). For *NR3C1*, *HSD11B2*, and *FKBP5*, each variable showed different degrees of methylation extent; most showed a moderate degree of methylation, though a few scores had very low (e.g., *HSD11B2*-6) and very high methylation extent (e.g., *HSD11B2*-7). Males had significantly lower methylation to CpGs on *HSD11B2*-3 compared to females (0.58 vs 0.60 respectively, *p* < 0.001). There were no other significant differences according to infant sex for GEA, PGES, *NR3C1*, *FKBP5*, or other *HSD11B2* scores.

Supplemental Tables S1–S3 list genomic location and methylation extent per CpG for each HPA-axis DNA methylation factor score. *NR3C1–1* and *NR3C1–2* CpGs were highly methylated largely located in the gene body and 5’UTR regions. *NR3C1–3* CpGs were low methylated and located in TSS1500. *NR3C1–5* and *NR3C1–6* CpGs with the highest loadings were located in the gene body and 5’UTR respectively. For *HSD11B2*, scores largely differentiated according to TSS and gene body locations, and most were low methylated. For *FKBP5–1*, CpGs were largely located in 5’UTR and the gene body (highly methylated), and *FKBP5–2* CpGs were located in gene body, 5’UTR, and TSS locations (moderately methylated). Both *FKBP5*-3 and *FKBP5*-4 had CpGs located in 5’UTR and TSS locations, with high (factor 3) and low (factor 4) methylation extents. *FKBP5*-5 included moderately methylated CpGs in the gene body. *FKBP5*-6 and *FKBP5*-7 both included a mix of CpGs from 5’UTR, body, and TSS locations, and were differentiated by methylation extent (factor 6 high; factor 7 low).

Table 2 shows the correlations among the epigenetic variables, adjusted for cell type proportions. Significant correlations were observed for GEA with PGES (*r* = −0.14, *p* = 0.04) and several HPA methylation scores including *NR3C1*-3 (*r* = 0.18, *p* = 0.01), *NR3C1*-4 (*r* = −0.21, *p* = 0.004), *NR3C1*-5 (*r* = −0.17, *p* = 0.02), and *HSD11B2*-3 (*r* = 0.18, *p* = 0.01), and *FKBP5*-3 (*r* = −0.20, *p* = 0.01). PGES was significantly correlated with some HPA methylation scores, including *NR3C1*-1 (*r* = 0.43, *p* < 0.001), *NR3C1*-6 (*r* = 0.38, *p* < 0.001), *HSD11B2*-3 (*r* = 0.32, *p* < 0.001), *HSD11B2*-7 (*r* = 0.43, *p* < 0.001), *FKBP5*-1 (*r* = 0.43, *p* < 0.001), and *FKBP5*-7 (*r* = 0.19, *p* = 0.01). The other 14 HPA scores were not correlated with PGES. Within and across HPA genes, some but not all methylation scores were significantly correlated with each other. The CpG sites that comprise the GEA, PGES, and HPA methylation scores are distinct and do not overlap (Supplemental file 1).

**Table 2.** Correlations among epigenetic variables.

		1	2	3	4	5	6	7	8	9	10	11	12	13	14	15	16	17	18	19	20	21	22
1	GEA	1.00																					
2	PGES	**−0.15**	1.00																				
3	NR3C1_1	−0.03	**0.43**	1.00																			
4	NR3C1_2	−0.06	−0.10	−0.11	1.00																		
5	NR3C1_3	**0.18**	0.01	−0.07	**0.19**	1.00																	
6	NR3C1_4	**−0.21**	−0.11	**−0.41**	**0.57**	**0.19**	1.00																
7	NR3C1_5	**−0.17**	−0.13	**−0.31**	**0.63**	**0.28**	**0.65**	1.00															
8	NR3C1_6	0.04	**0.38**	**0.79**	−0.09	−0.09	**−0.36**	**−0.23**	1.00														
9	HSD11B2_1	−0.10	0.11	0.15	−0.04	0.03	−0.08	**−0.13**	−0.02	1.00													
10	HSD11B2_2	0.06	0.06	0.09	**−0.15**	−0.05	**−0.17**	−0.12	0.07	0.10	1.00												
11	HSD11B2_3	**0.18**	**0.32**	**0.36**	−0.13	0.11	**−0.20**	**−0.18**	**0.25**	0.02	−0.06	1.00											
12	HSD11B2_4	−0.02	0.05	0.03	0.07	−0.05	0.11	0.02	**0.16**	0.11	**0.15**	0.02	1.00										
13	HSD11B2_5	−0.01	0.06	−0.12	0.14	0.25	0.13	**0.19**	−0.03	0.06	−0.04	0.03	**0.15**	1.00									
14	HSD11B2_6	−0.09	−0.08	0.06	−0.06	−0.01	−0.04	−0.03	0.08	**0.30**	0.03	−0.07	0.12	0.08	1.00								
15	HSD11B2_7	−0.08	**−0.28**	**−0.37**	0.10	0.06	**0.22**	**0.20**	**−0.40**	−0.05	0.06	**−0.15**	−0.07	−0.14	0.03	1.00							
16	FKBP5_1	0.08	**0.43**	**0.86**	−0.07	0.02	**−0.40**	**−0.33**	**0.72**	0.08	0.04	**0.38**	−0.05	−0.12	−0.01	**−0.36**	1.00						
17	FKBP5_2	0.07	0.07	0.05	**0.16**	0.13	0.14	**0.15**	−0.08	0.01	0.05	**0.15**	−0.02	0.05	0.03	−0.03	−0.04	1.00					
18	FKBP5_3	**−0.20**	−0.04	−0.07	**0.19**	−0.12	0.13	0.13	−0.03	−0.12	0.07	−0.05	−0.01	−0.10	0.04	0.14	0.02	0.07	1.00				
19	FKBP5_4	−0.12	0.12	0.09	−0.09	−0.01	**−0.10**	−0.10	**0.17**	**0.37**	0.12	0.07	0.12	**0.28**	**0.45**	−0.05	0.04	0.05	−0.03	1.00			
20	FKBP5_5	0.01	0.07	−0.04	0.13	**0.15**	0.11	**0.18**	−0.07	0.02	0.04	−0.02	−0.02	**0.27**	0.00	−0.05	−0.01	−0.01	0.04	0.02	1.00		
21	FKBP5_6	−0.08	0.06	**0.15**	**0.40**	0.02	0.08	**0.23**	0.05	0.04	−0.02	0.05	0.09	0.14	0.12	−0.04	0.08	**0.14**	0.08	0.12	**0.20**	1.00	
22	FKBP5_7	−0.12	**0.19**	**0.43**	−0.11	−0.14	**−0.18**	**−0.16**	**0.27**	0.08	0.05	**0.23**	0.11	−0.05	0.10	−0.12	**0.35**	0.01	−0.11	0.13	−0.01	**0.20**	1.00

Bold cell entries are significant a *p* < 0.05. Controlled for cell type proportions (Bcell, CD4T, CD8T, Gran, Mono, NK, nRBC). GEA=Gestational epigenetic aging; PGES=Polygenetic glucocorticoid exposure score.

Table 3 lists the multivariable linear regression models for the associations between PGES and HPA methylation scores with GEA for the full sample and also stratified by infant sex. In the full sample, we found that PGES was associated with gestational epigenetic age deceleration (β=-1.12, SE = 0.047, *p* = 0.02), controlling for cell type and other covariates. Similarly, *NR3C1* scores 1, 4, 5, and also *FKBP5* scores 1 and 3 were each negatively associated with GEA (all *p* < 0.05). *NR3C1*-3 was positively associated with GEA (β = 0.22, SE = 0.08, *p* = 0.004). Non-significant positive trends were observed for *HSD11B2*-3 (β = 0.16, SE = 0.08, *p* = 0.06) and *FKBP5*-1 (β = 0.14, SE = 0.08, *p* = 0.09) with GEA.

**Table 3.** Multivariable linear regression associations between the polyepigenetic glucocorticoid exposure score, hpa-axis DNA methylation scores, and gestational epigenetic age deviation*.

	Full sample		Males		Females	
	β (SE)	p	β (SE)	p	β (SE)	p
PGES	−1.12 (0.47)	0.02	−0.91 (0.67)	0.18	−1.11 (0.71)	0.12
*NR3C1* − 1	−0.35 (0.14)	0.01	−0.23 (0.24)	0.34	−0.28 (0.21)	0.19
*NR3C1* − 2	0.26 (0.18)	0.14	0.37 (0.37)	0.31	−0.10 (0.26)	0.70
*NR3C1* − 3	0.22 (0.08)	0.004	0.21 (0.13)	0.10	0.13 (0.11)	0.27
*NR3C1* − 4	−0.28 (0.12)	0.02	−0.20 (0.19)	0.33	−0.37 (0.17)	0.04
*NR3C1* − 5	−0.28 (0.14)	0.04	−0.45 (0.19)	0.02	0.15 (0.25)	0.55
*NR3C1* − 6	0.18 (0.13)	0.16	0.05 (0.24)	0.84	0.19 (0.19)	0.32
*HSD11B2* − 1	−0.11 (0.08)	0.17	−0.15 (0.12)	0.22	−0.01 (0.12)	0.91
*HSD11B2* − 2	0.07 (0.08)	0.39	0.01 (0.13)	0.90	0.08 (0.11)	0.46
*HSD11B2* − 3	0.16 (0.08)	0.06	0.17 (0.12)	0.14	0.18 (0.13)	0.18
*HSD11B2* − 4	0.01 (0.08)	0.93	0.22 (0.13)	0.10	−0.14 (0.11)	0.19
*HSD11B2* − 5	0.01 (0.08)	0.91	0.15 (0.12)	0.23	−0.16 (0.11)	0.14
*HSD11B2* − 6	−0.04 (0.08)	0.59	−0.10 (0.14)	0.48	−0.02 (0.11)	0.88
*HSD11B2* − 7	−0.06 (0.08)	0.39	0.16 (0.13)	0.22	−0.16 (0.10)	0.12
*FKBP5* − 1	0.14 (0.08)	0.09	0.06 (0.12)	0.65	0.20 (0.14)	0.15
*FKBP5* − 2	0.26 (0.18)	0.13	0.41 (0.30)	0.17	0.14 (0.23)	0.54
*FKBP5* − 3	−0.28 (0.08)	0.004	−0.45 (0.15)	0.007	−0.17 (0.11)	0.14
*FKBP5* − 4	−0.12 (0.07)	0.10	−0.11 (0.13)	0.39	−0.09 (0.10)	0.34
*FKBP5* − 5	0.03 (0.07)	0.70	0.02 (0.10)	0.81	0.08 (0.13)	0.54
*FKBP5* − 6	−0.08 (0.15)	0.62	−0.14 (0.25)	0.57	−0.10 (0.22)	0.66
*FKBP5* − 7	−0.16 (0.08)	0.04	−0.20 (0.11)	0.06	−0.12 (0.14)	0.41

*All models were adjusted for maternal age, race/ethnicity, education, pre-pregnancy BMI, parity, pregnancy conditions, smoking, diet, depressive symptoms, and cell type proportions (Bcell, CD4T, CD8T, Gran, Mono, NK, nRBC). Analyses among the full sample additionally adjust for infant sex.

HPA=Hypothalamic pituitary adrenal; PGES=Polyepigenetic glucocorticoid exposure score.

When stratifying by infant sex, higher levels of methylation on *NR3C1–5* (β=-0.45 SE = 0.19, *p* = 0.02) and *FKBP5–3* (β=-0.45, SE = 0.15, *p* = 0.007) were negatively associated with GEA among male but not female infants. A non-significant trend suggested *FKBP5*-7 May also be associated with decelerated aging among males (β =-0.20, SE = 0.11, *p* = 0.06) and not females. For females, higher *NR3C1–4* scores were significantly associated with decelerated aging (β=-0.37, SE = 0.17, *p* = 0.04).

**Figure 1.**
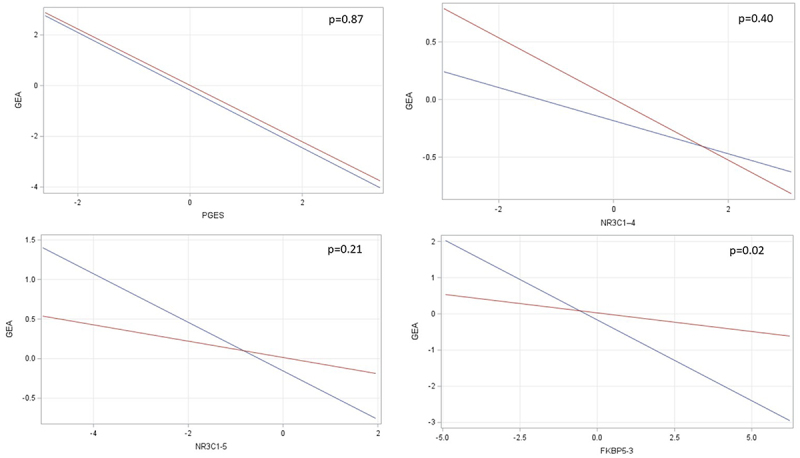
Interaction plots for the association between PGES and DNA methylation factor scores with gestational epigenetic aging according to infant sex. Slopes for males appear in Blue; slopes for females appear in red. P-values list the significance level for the interaction effect.

For the epigenetic factors that demonstrated significant sex-specific associations (*p* < 0.05) with GEA in stratified models (*NR3C1–5*, *NR3C1–4*, and *FKBP5–3*), we fit additional models for GEA that considered the statistical interaction between that predictor with infant sex (Figure 1). Also, in an effort to replicate prior work where sex-specific patterning in PGES and GEA associations was observed [23], we tested for interaction effect for PGES and GEA despite observing no sex patterning in our stratified models. Of these, the interaction effect was significant for *FKBP5–3* (β =-0.34, SE = 0.15, *p* = 0.02), whereby the GEA slope was relatively flat for females and decelerated for males. No interaction effect was observed for PGES (β = 0.03, SE = 0.16, *p* = 0.87); the PGES slopes were parallel and nearly coincident for male and females. No significant interaction effects were observed for *NR3C1–4* or *NR3C1–5*, though the plots suggest the possibility of differential patterning in GEA by sex.

## Discussion

DOHaD research has revealed the sensitivity of the fetal epigenome to gestational exposures, including for GEA. This study focused on elucidating molecular mechanisms that may help explain how psychosocial adversity during pregnancy might influence epigenetic processes *in utero*. We found that gestational DNA methylation to glucocorticoid sensitive CpGs and HPA-axis genes were associated with deviations in GEA. We replicated the association between PGES and decelerated GEA in an independent sample, as well as identified a novel set of *NR3C1* and *FKBP5* markers that were similarly associated with GEA. Some sex-specific patterning observed in these associations. This study provides new insights into how molecular markers of glucocorticoid regulation and psychosocial adversity during pregnancy may contribute to gestational epigenetic aging signatures at birth.

Euclydes et al [23]. demonstrated that PGES was negatively associated with GEA in sample of 83 mother and infant pairs from Brazil. We similarly observed decelerated GEA in association with higher PGES scores. This replication is notable, as we tested study hypotheses in a larger and independent sample in the United States, with broader racial, ethnic, and socioeconomic diversity. In addition, we identified novel sets of CpGs for *NR3C1* (factors 4 and 5) and *FKBP5* (factors 3 and 7) where higher levels of DNA methylation were similarly negatively associated with GEA. For *NR3C1* (factors 4 and 5), CpGs were moderately to highly methylated and located in both promoter and gene body locations. For *FKBP5*, CpGs were had either moderate (factor 3) or low degrees of methylation extent (factor 7) and were largely located in 5’UTR regions. These findings suggest that patterning in methylation extent in these regions may influence dysregulation of the developing fetal HPA axis system, possibly through transcriptional silencing and modulation of gene expression, which in turn may negatively influences GEA at birth. The only set of CpG sites positively associated with GEA were for *NR3C1* factor 3, where CpGs were unmethylated at TSS start sites. These associations are consistent with work from other cohorts showing maternal adversity can influence HPA axis gene methylation extent [10] and deviations in GEA at birth [16–19]. Thus, our findings suggest that decelerated GEA at birth may be attributable in part to methylation extent of glucocorticoid sensitive CpG sites and HPA axis genes.

We observed sex patterning in GEA across a set of *NR3C1* and *FKBP5* factors. The most robust association was observed for *FKBP5* factor 3, where a negative association with GEA was observed for males but not females in stratified models and when testing for an interaction effect. This pattern is consistent with the DOHaD framework that underscores sex differences in developmental programming [42], and also consistent with empirical work showing sex specific associations in gestational epigenetic alterations to glucocorticoid response genes (including *FKPB5*), GEA, and neurobehavioral problems for males exposed to psychosocial risks in utero [11,16]. However, we observed no sex-specific patterning in PGES and GEA, which is in contrast to Euclydes et al [23] that showed significant associations for female infants. Methodological differences between the two studies could account for this discrepancy in findings, including controlling for different sets of maternal health and behavioral covariates and lower power to detect effects among males in Euclydes (*n* = 37 male infants) relative to AIMS (*n* = 99 male infants). Male and female fetuses may respond differently to prenatal psychosocial exposures, due in part to differences in hormonal environments, maternal behavioral factors, and health conditions that together may influence GEA signatures [43]. As our study provides partial support for sex-specific patterning in glucocorticoid regulation related DNA methylation and GEA, we encourage future work to continue to evaluate this possibility.

It was surprising that *HSD11B2* methylation scores were not significantly associated with GEA in any model, as prior work has linked gestational *HSD11B2* methylation extent to prenatal psychosocial adversity [12,44], and to infant neurobehavioral outcomes [13]. Low methylation extent was observed for 16 of the 23 CpGs (70%), and this constrained variability could have led to null or underestimated associations with GEA in our models. An alternative interpretation of the null findings could be related to the function of the HSD11B2 gene and its adaptive process in HPA axis development during gestation. The HSD11B2 gene encodes the 11-beta hydroxysteroid dehydrogenase enzyme, which is responsible for the inactivation of maternal cortisol thereby protecting the developing fetus from over exposure to stress hormones during development [45]. In the absence of significant methylation extent to its CpGs, HSD11B2 may be functioning optimally and thus would not be expected to contribute to a risky GEA phenotype like decelerated aging. As we did not measure gene expression and cannot test this possibility explicitly, we encourage future work to examine this association and consider the role of HSD11B2 expression in gestational epigenetic aging.

This study had some limitations. First, we used the Knight [7] clock to estimate GEA which while validated for use in cord blood, has been critiqued for its precision [46] relative to other GEA measures including a metric derived with EPIC array data [47]. We acknowledge the estimates we present here may be imprecise. We used the Knight clock in order to facilitate comparisons to our prior GEA work in this sample [18] and with other recent GEA studies focused on prenatal exposures from major birth cohorts [16,17,48,49]. We encourage future work replicate this study and incorporate additional and measures of GEA to enhance the precision of the estimates. Also, participants missing umbilical cord blood samples and methylation information were excluded from analysis. These participants were more likely to be racial and ethnic minorities and low socioeconomic status, which in turn may have constrained variability in methylation extent and led to underestimated associations. Finally, as we did not test whether the PGES, HPA-related DNA methylation, and GEA was associated with infant outcomes, it is not known whether associations reported in this analysis indicate child health risk. We encourage future research to examine these methylation variables in relation to infant phenotypes.

This study also had strengths. First, we used a multimodal design that integrated information from umbilical cord blood derived DNA methylation with questionnaire and hospital medical records. Also, we took steps to reduce the risk of type 1 error though applying a data reduction technique to reduce the dimensionality of the DNA methylation information into a smaller set of variables. Moreover, there was no overlap or duplication in CpGs across the PGES and the HPA axis factor scores, and correlations among the methylation variables were low. This indicates that PGES, *NR3C1*, and *FKBP5* methylation scores each explained unique variability in GEA. Finally, our multivariate models had robust confounding control as we included a critical set of demographic factors, maternal health, and behavioral factors.

Gestational epigenetic aging is a novel approach for characterizing the potential impacts of prenatal exposures and researchers are just beginning to understand its determinants and implications for postnatal life. In this study, we found that methylation extent to a set of CpGs sensitive synthetic dexamethasone exposure and to genes involved in glucocorticoid regulation was associated with GEA signatures at birth, including decelerated aging that might indicate a neonatal risk phenotype. As the field of epigenetic aging continues to mature, we encourage researchers to link population-based exposures and DNA methylation mechanisms with GEA to fully understand how GEA might influence postnatal health. Doing so may yield new insights on the molecular and developmental origins of health and disease, and also offer novel perspectives for interdisciplinary intervention efforts for pregnant women and infants.

## Supplementary Material

_supplemental tables_08212024.xlsx

## Data Availability

The participants of this study did not give written consent for their data to be shared publicly, so due to the sensitive nature of the research supporting data is not available.

## References

[1] Barker DJ. In utero programming of chronic disease. Clin Sci. 1998;95(2):115–12. doi: 10.1042/cs0950115 9680492

[2] Hoffman DJ, Powell TL, Barrett ES, et al. Developmental origins of metabolic diseases. Physiol Rev. 2021;101(3):739–795. doi: 10.1152/physrev.00002.2020 33270534 PMC8526339

[3] Lapehn S, Paquette AG. The placental epigenome as a molecular link between prenatal exposures and fetal health outcomes through the DOHaD hypothesis. Curr Environ Health Rep. [cited 2022 Apr 29];9(3):490–501. doi: 10.1007/s40572-022-00354-8 35488174 PMC9363315

[4] Doi M, Usui N, Shimada S. Prenatal environment and neurodevelopmental disorders. Front Endocrinol (Lausanne). 2022;13:860110. doi: 10.3389/fendo.2022.860110 35370942 PMC8964779

[5] Clark J, Bulka CM, Martin CL, et al. Placental epigenetic gestational aging in relation to maternal sociodemographic factors and smoking among infants born extremely preterm: a descriptive study. Epigenetics. 2022;17(13):2389–2403. doi: 10.1080/15592294.2022.2125717 36134874 PMC9665142

[6] Horvath S, Raj K. DNA methylation-based biomarkers and the epigenetic clock theory of ageing. Nat Rev Genet. 2018;19(6):371–384. doi: 10.1038/s41576-018-0004-3 29643443

[7] Knight AK, Craig JM, Theda C, et al. An epigenetic clock for gestational age at birth based on blood methylation data. Genome Biol. 2016;17(1):206. doi: 10.1186/s13059-016-1068-z 27717399 PMC5054584

[8] Sosnowski DW, Booth C, York TP, et al. Maternal prenatal stress and infant DNA methylation: a systematic review. Dev Psychobiol. 2018;60(2):127–139. doi: 10.1002/dev.21604 29344930

[9] Chalfun G, Araújo Brasil AD, Paravidino VB, et al. NR3C1 gene methylation and cortisol levels in preterm and healthy full-term infants in the first 3 months of life. Epigenomics. 2022;14(24):1545–1561. doi: 10.2217/epi-2022-0444 36861354

[10] Azar N, Booij L. DNA methylation as a mediator in the association between prenatal maternal stress and child mental health outcomes: current state of knowledge. J Affect Disord. 2022;319:142–163. doi: 10.1016/j.jad.2022.09.008 36113690

[11] Liu H, Liu Y, Huang K, et al. Gender-specific associations of pregnancy-related anxiety with placental epigenetic patterning of glucocorticoid response genes and preschooler’s emotional symptoms and hyperactivity. BMC Pediatr. 2021;21(1):479. doi: 10.1186/s12887-021-02938-z 34715840 PMC8555194

[12] Appleton AA, Armstrong DA, Lesseur C, et al. Patterning in placental 11-B hydroxysteroid dehydrogenase methylation according to prenatal socioeconomic adversity. PLOS ONE. 2013;8(9):e74691. doi: 10.1371/journal.pone.0074691 24040322 PMC3764127

[13] Appleton AA, Lester BM, Armstrong DA, et al. Examining the joint contribution of placental NR3C1 and HSD11B2 methylation for infant neurobehavior. Psychoneuroendocrinology. 2015;52:32–42. doi: 10.1016/j.psyneuen.2014.11.004 25459891 PMC4350656

[14] Grasso DJ, Drury S, Briggs-Gowan M, et al. Adverse childhood experiences, posttraumatic stress, and FKBP5 methylation patterns in postpartum women and their newborn infants. Psychoneuroendocrinology. 2020;114:104604. doi: 10.1016/j.psyneuen.2020.104604 32109789 PMC7096279

[15] Oberlander TF, Weinberg J, Papsdorf M, et al. Prenatal exposure to maternal depression, neonatal methylation of human glucocorticoid receptor gene (NR3C1) and infant cortisol stress responses. Epigenetics. 2008;3(2):97–106. doi: 10.4161/epi.3.2.6034 18536531

[16] Suarez A, Lahti J, Czamara D, et al. The epigenetic clock at birth: associations with maternal antenatal depression and child psychiatric problems. J Am Acad Child Adolesc Psychiatry. 2018;57(5):321–328 e2. doi: 10.1016/j.jaac.2018.02.011 29706161 PMC6277971

[17] McKenna BG, Hendrix CL, Brennan PA, et al. Maternal prenatal depression and epigenetic age deceleration: testing potentially confounding effects of prenatal stress and SSRI use. Epigenetics. 2021;16(3):327–337. doi: 10.1080/15592294.2020.1795604 32660321 PMC7901550

[18] Appleton AA, Lin B, Kennedy EM, et al. Maternal depression and adverse neighbourhood conditions during pregnancy are associated with gestational epigenetic age deceleration. Epigenetics. 2022;17(13):1905–1919. doi: 10.1080/15592294.2022.2090657 35770941 PMC9665127

[19] McGill MG, Pokhvisneva I, Clappison AS, et al. Maternal prenatal anxiety and the fetal origins of epigenetic aging. Biol Psychiatry. 2022;91(3):303–312. doi: 10.1016/j.biopsych.2021.07.025 34756561

[20] Knight AK, Smith AK, Conneely KN, et al. Relationship between epigenetic maturity and respiratory morbidity in preterm infants. J Pediatr. 2018;198:168–173.e2. doi: 10.1016/j.jpeds.2018.02.074 29705119 PMC6261285

[21] Provençal N, Arloth J, Cattaneo A, et al. Glucocorticoid exposure during hippocampal neurogenesis primes future stress response by inducing changes in DNA methylation. Proc Natl Acad Sci. 2020;117(38):23280–23285. doi: 10.1073/pnas.1820842116 31399550 PMC7519233

[22] Suarez A, Lahti J, Lahti-Pulkkinen M, et al. A polyepigenetic glucocorticoid exposure score at birth and childhood mental and behavioral disorders. Neurobiol Stress. 2020;13:100275. doi: 10.1016/j.ynstr.2020.100275 33344728 PMC7739178

[23] Euclydes V, Gomes C, Gouveia G, et al. Gestational age acceleration is associated with epigenetic biomarkers of prenatal physiologic stress exposure. Clin Epigenetics. 2022;14(1):152. doi: 10.1186/s13148-022-01374-9 36443840 PMC9703828

[24] Versteegen M, Bozlak CT, Larkin H, et al. Maternal depression, adverse childhood experiences, and social support in relation to gestational diabetes risk: results from the Albany infant and mother study (AIMS). BMC Pregnancy Childbirth. 2021;21(1):335. doi: 10.1186/s12884-021-03814-5 33906618 PMC8077784

[25] Appleton AA, Lin B, Holdsworth EA, et al. Prenatal exposure to favorable social and environmental neighborhood conditions is associated with healthy pregnancy and infant outcomes. Int J Environ Res Public Health. 2021;18(11):6161. doi: 10.3390/ijerph18116161 34200387 PMC8200992

[26] Appleton AA, Kiley K, Holdsworth EA, et al. Social support during pregnancy modifies the association between maternal adverse childhood experiences and infant birth size. Matern Child Health J. 2019;23(3):408–415. doi: 10.1007/s10995-018-02706-z 30627949

[27] Appleton AA, Kiley KC, Schell LM, et al. Prenatal lead and depression exposures jointly influence birth outcomes and NR3C1 DNA methylation. Int J Environ Res Public Health. 2021;18(22):12169. doi: 10.3390/ijerph182212169 34831923 PMC8620070

[28] Moran S, Arribas C, Esteller M. Validation of a DNA methylation microarray for 850,000 CpG sites of the human genome enriched in enhancer sequences. Epigenomics. 2016;8(3):389–399. doi: 10.2217/epi.15.114 26673039 PMC4864062

[29] Aryee MJ, Jaffe AE, Corrada-Bravo H, et al. Minfi: a flexible and comprehensive bioconductor package for the analysis of infinium DNA methylation microarrays. Bioinformatics. 2014;30(10):1363–1369. doi: 10.1093/bioinformatics/btu049 24478339 PMC4016708

[30] Pidsley R, Y Wong CC, Volta M, et al. A data-driven approach to preprocessing illumina 450K methylation array data. BMC Genomics. 2013;14:293. doi: 10.1186/1471-2164-14-293 23631413 PMC3769145

[31] Johnson WE, Li C, Rabinovic A. Adjusting batch effects in microarray expression data using empirical Bayes methods. Biostatistics. 2007;8(1):118–127. doi: 10.1093/biostatistics/kxj037 16632515

[32] Leek J, Johnson W, Parker H, et al. Sva: surrogate variable analysis. 2021.

[33] Xu Z, Niu L, Taylor JA. The ENmix DNA methylation analysis pipeline for illumina BeadChip and comparisons with seven other preprocessing pipelines. Clin Epigenetics. 2021;13(1):216. doi: 10.1186/s13148-021-01207-1 34886879 PMC8662917

[34] McEwen LM, Jones MJ, Lin DTS, et al. Systematic evaluation of DNA methylation age estimation with common preprocessing methods and the infinium MethylationEPIC BeadChip array. Clin Epigenetics. 2018;10(1):123. doi: 10.1186/s13148-018-0556-2 30326963 PMC6192219

[35] Paquette AG, Lester BM, Lesseur C, et al. Placental epigenetic patterning of glucocorticoid response genes is associated with infant neurodevelopment. Epigenomics. 2015;7(5):767–779. doi: 10.2217/epi.15.28 26343289 PMC4772971

[36] Elovitz MA, Anton L, Bastek J, et al. Can microRNA profiling in maternal blood identify women at risk for preterm birth? Am J Obstet Gynecol. 2015;212(6):e782.1–e782.5. doi: 10.1016/j.ajog.2015.01.023 25617732

[37] Horn JL. A rationale and test for the number of factors in factor analysis. Psychometrica. 1965;30(2):179–185. doi: 10.1007/BF02289447 14306381

[38] Ledesma DR, Mora PV. Determining the number of factors to retain in EFA: an easy-to-use computer program for carrying out parallel analysis. Practica Assess, Res & Eval. 2007;12(2):1–11.

[39] Cox JL, Holden JM, Sagovsky R. Detection of postnatal depression: development of the 10-item edinburgh postnatal depression scale. Br J Psychiatry: J Ment Sci. 1987;150(6):782–786. doi: 10.1192/bjp.150.6.782 3651732

[40] Michaud DS, Skinner HG, Wu K, et al. Dietary patterns and pancreatic cancer cancer risk in men and women. J Natl Cancer Inst. 2005;97(7):518–524. doi: 10.1093/jnci/dji094 15812077

[41] Willett WC. Nutritional epidemiology. 2nd ed. New York: Oxford University Press; 1998.

[42] Sundrani DP, Roy SS, Jadhav AT, et al. Sex-specific differences and developmental programming for diseases in later life. Reprod Fertil Dev. 2017;29(11):2085–2099. doi: 10.1071/RD16265 28380326

[43] Girchenko P, Lahti J, Czamara D, et al. Associations between maternal risk factors of adverse pregnancy and birth outcomes and the offspring epigenetic clock of gestational age at birth. Clin Epigenetics. 2017;9(1):49. doi: 10.1186/s13148-017-0349-z 28503212 PMC5422977

[44] Conradt E, Lester BM, Appleton AA, et al. The roles of DNA methylation of NR3C1 and 11beta-HSD2 and exposure to maternal mood disorder in utero on newborn neurobehavior. Epigenet: Off J DNA Methylation Soc. 2013;8(12):1321–1329. doi: 10.4161/epi.26634 PMC3933492 24135662

[45] Staud F, Mazancová K, Miksík I, et al. Corticosterone transfer and metabolism in the dually perfused rat placenta: effect of 11β-hydroxysteroid dehydrogenase type 2. Placenta. 2006;27(2–3):171–180. doi: 10.1016/j.placenta.2005.01.001 16338462

[46] Simpkin AJ, Suderman M, Howe LD. Epigenetic clocks for gestational age: statistical and study design considerations. Clin Epigenetics. 2017;9(1):100. doi: 10.1186/s13148-017-0402-y 28932320 PMC5602851

[47] Haftorn KL, Lee Y, Denault WRP, et al. An EPIC predictor of gestational age and its application to newborns conceived by assisted reproductive technologies. Clin Epigenetics. 2021;13(1):82. doi: 10.1186/s13148-021-01055-z 33875015 PMC8056641

[48] Dye CK, Alschuler DM, Wu H, et al. Maternal adverse childhood experiences and biological aging during pregnancy and in newborns. JAMA Netw Open. 2024;7(8):e2427063. doi: 10.1001/jamanetworkopen.2024.27063 39120899 PMC11316241

[49] Ladd-Acosta C, Vang E, Barrett ES, et al. Analysis of pregnancy complications and epigenetic gestational age of newborns. JAMA Netw Open. 2023;6(2):e230672. doi: 10.1001/jamanetworkopen.2023.0672 36826815 PMC9958528

